# The relativity of Darwinian populations and the ecology of endosymbiosis

**DOI:** 10.1007/s10539-016-9531-5

**Published:** 2016-06-14

**Authors:** Adrian Stencel

**Affiliations:** Faculty of Philosophy, Jagiellonian University, Gołębia 24, 31-007 Kraków, Poland

**Keywords:** Endosymbiosis, Population, Relativity, Transitions

## Abstract

If there is a single discipline of science calling the basic concepts of biology into question, it is without doubt microbiology. Indeed, developments in microbiology have recently forced us to rethink such fundamental concepts as the organism, individual, and genome. In this paper I show how microorganisms are changing our understanding of natural aggregations and develop the concept of a Darwinian population to embrace these discoveries. I start by showing that it is hard to set the boundaries of a Darwinian population, and I suggest thinking of a Darwinian population as a relative property of a Darwinian individual. Then I argue, in contrast to the commonly held view, that Darwinian populations are multispecies units, and that in order to accept the multispecies account of Darwinian populations we have to separate fitness from natural selection. Finally, I show how all these ideas provide a theoretical framework leading to a more precise understanding of the ecology of endosymbiosis than is afforded by poetic metaphors such as ‘slavery’.

## Introduction

Population thinking is embedded in the foundation of the modern theory of natural selection (Mayr 1959; Godfrey-Smith 2009). We do not study evolution irrespective of populations. Whether our object of study is a gene, an individual species, or even a clade, there is always a relationship to entities of the same or a similar class. Despite this obvious fact and suggestions from biologists calling for ‘a reexamination of the very concepts of what constitutes a genome, a population, an environment, and an organism’(McFall-Ngai et al. 2013), due to recent developments in microbiology, the concept of population has not been rethought as those of organism (Sleator 2010; Booth 2014) or genome (Zilber-Rosenberg and Rosenberg 2008; Stencel and Crespi 2013) have been. However, it really ought to be, as microorganisms are changing our understanding of natural aggregations. For example, it has been reported that the human gut contains more than 1000 bacterial species (Rajilic-Stojanovic et al. 2007) and that the number of bacteria in the human body is approximately 10 times the number of human cells (Kurokawa et al. 2007) disproved the idea that humans interact mostly with animals and plants and only occasionally with microbes, such as influenza germs, that kill them. The picture emerging from ongoing research is rather the opposite: humans interact mainly with microorganisms and only ‘occasionally’ with animals and plants. How, then, can we conceptualise a population to incorporate into a single idea entities such as microorganisms and animals placed upon such distant branches of the tree of life?

There is only one theory with the capacity to unify biology in its entirety: the theory of evolution by natural selection. A general concept of a population, therefore, has to be developed under the banner of the theory of evolution. Interestingly, some steps toward this unification have been taken. Peter Godfrey-Smith, in his seminal work Darwinian populations and Natural Selection (2009), developed a concept of Darwinian population which was supposed to serve as a universal tool in explaining the process of evolution, from the evolution of wild populations through evolutionary transitions to cultural change. However, he did not spend much time showing how to look, using his framework, at multi-species Darwinian populations. The aim of this paper is to show, therefore, that Darwinian populations are, in fact, multi-species communities and that a reproducer`s framework may be a useful tool to understand their evolution. The paper is organised as follows. Firstly, I argue, following Godfrey-Smith (2009), that reproducers are units of selection. Then I show that it might be very hard to distinguish (in accordance with the teachings of microbiology) a Darwinian population by taking a group of individuals as a starting point. Accordingly, I suggest thinking of a Darwinian population as a relative property of a Darwinian individual—a set of reproducers that engages in fitness-affecting interactions with a focal unit. Then I argue that there is nothing wrong with a multi-species account of a Darwinian population as long as we distinguish Darwinian interactions from fitness. Finally, I show how these ideas might be useful in understanding the ecology of endosymbiosis. Mainly, I show that the ecology of endosymbiosis might be understood in better terms than poetic metaphors such as ‘slavery’(Maynard-Smith and Szathmary 1995).

## Reproducers as units of selection

Lakatos (1970) argued that scientific progress is made through competition between and eventually substitution of research programmes, that is, sets of interconnected theories which designate directions of research and the way research itself is conducted. If so, then there are two great research programmes involved in debates over levels of selection. The first is the result of the work of Dawkins (1976, 1982) and Hull (1980). In their framework, the focal unit of evolution is the replicator (for instance DNA), which transmits its structure intact from generation to generation. Different forms of the proliferation of replicators across generations are caused by interactors (in Hull’s terminology), or by vehicles (in Dawkins’s), that is, by temporal entities produced by replicators in every successive generation, constituting their machines of survival. Thus, for evolution to operate through natural selection, replicators, which pass down their structures from generation to generation, and interactors/vehicles, which make certain replicators more or less common across generations, are required. The second research programme, less demanding but more generally useful, was first formalised by Lewontin (1970). Here I follow the version developed by Godfrey-Smith (2009).

My starting point is evolution through natural selection (ENS). A population which can undergo ENS must comprise causally connected objects (called here Darwinian individuals), which must in turn be characterised by three properties. Firstly, they must vary with respect to traits such as weight, intelligence or biochemical pathways. Furthermore, these traits must influence the fitness output of the Darwinian individuals. This means that differences in traits influence the reproduction and survival of individuals. Finally, these traits must be heritable, at least to some extent, by offspring. Evolution by natural selection, thus, will take place in any population in which there are phenotypic variations, heritability, and differences in fitness (reproductive output) caused, at least in part, by these variations. Combining these three properties, we come to the conclusion that Darwinian individuals must be reproducers (Griesemer 2000; Godfrey-Smith 2009), because reproducers are the only units capable of transforming the resources they find around them in order to produce more reproducers and thus are causally responsible for parent-offspring similarity (fulfilling the heritability criterion mentioned above). Assuming we have, therefore, a diverse population of reproducers competing for limited resources, only some reproducers will obtain them and produce offspring; others will undergo decay, gradually transforming them back into resources: the thread which has linked them via parent-offspring lineage to the first reproducer is broken. Indeed, such a population will experience evolution by natural selection.

Interestingly, we find different kinds of reproducers in nature. Godfrey-Smith (2009) cited three. The first are scaffolded reproducers: those entirely dependent on external machinery. For instance, viruses belong to this category, because they need the biochemical machinery of cells to reproduce. The second category includes simple reproducers. Reproducers of this kind possess inner machinery, and thus need only external resources to initiate reproduction. One example is a bacterial cell. The third category constitutes collective reproducers, i.e. those built of simple reproducers. In essence, a collective reproducer is an entity that can reproduce itself, but which is also built of elements that can reproduce themselves. An example is multicellular individuals built of eukaryotic cells. The above classification is not artificial, but rather a consequence of the fact that evolution is a process not only of differential reproduction of Darwinian individuals, but as well one that leads to the origin of new kinds of Darwinian individuals, called transitions in individuality (Michod 2000; Godfrey-Smith 2009). These transitions occur when a group of units comes together to form a higher level of unit that can undergo the process of natural selection itself, as it becomes a new, collective reproducer, as in the case of transition from unicellular to multicellular individuals (Buss 1988; Maynard-Smith and Szathmary 1995) or from prokaryotic cells to eukaryotic cells containing mitochondria that were formerly free-living cells (Margulis 1981). Of course, this is not the whole story. Reproducers are much more diverse than the above classification suggests. This diversity was extensively elaborated by Godfrey-Smith (2009). For instance, collective reproducers such as animals and plants differ in their method of reproduction, since some species multiply sexually, some through fragmentation of the body and others via both routes. Such considerations are quite interesting and illuminating; however, for the purpose of this paper, the classification introduced above is sufficient.

## The relativity of Darwinian populations

In the above considerations is a hidden assumption. Godfrey-Smith (2009) (like many other researchers; see Lewontin 1970, Ridley 1996) began his considerations of the process of natural selection by assuming the existence of a population of causally connected individuals in which variation in character leads to differences in reproductive output and is inherited to some extent. At the beginning, therefore, he supposed that we are able to distinguish such a population from a mere aggregation of things. What does this mean? At first glance, one may think it means that Darwinian populations are objectively existing groups. Indeed, being a Darwinian population is a property of a group. We can take a group of things and argue that it is a Darwinian population because it fulfils certain criteria.

**Fig. 1:**
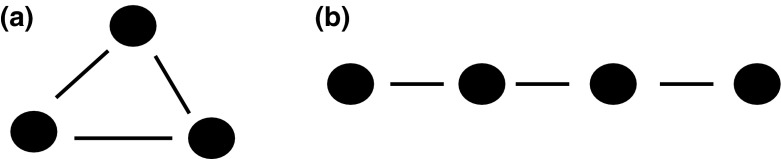
Two kinds of population structure: group structure (**a**) and neighbour structure (**b**). A *line* between two units indicates that they engage in fitness-affecting interactions

What criteria must a group fulfil to be called a Darwinian population? Basically, if we take a group of different Darwinian individuals with varying traits, for us to consider them as a Darwinian population they must engage in causal interactions (fitness-affecting interactions). In other words, if we have a group of three Darwinian individuals (A, B, and C), then A must be linked causally with B and C, B with A and C, and C with A and B (Fig. 1a). We can call such a group Darwinian because each member interacts with the others in a way that influences its fitness. Thus, it seems that Darwinian populations are discrete units that can easily be isolated from nature. This statement seems obvious and by no means controversial. After all, the essence of Darwinism is competition among members of a group. However, I do not believe this is entirely true and neither, I suspect, does Godfrey-Smith; he himself (2006) put forward some objections arguing that in the case of a neighbour-structured population, one cannot distinguish a population of interacting individuals so simply; the situation in such populations is too complex for that. Let me recall his arguments to show why it might be misleading to take a collection of reproducers as a starting point.

Consider that there is a group of four reproducers, A, B, C, and D, and that their fitness is causally connected, that is, A influences the reproductive output of B, B influences the reproductive output of C, and C influences the reproductive output of D. Based on these causal connections, one can infer that these reproducers constitute a Darwinian population, because interactions among these reproducers lead to differences in reproductive output in the population. However, this would be overreaching, since there is no causal connection between A and C or between B and D or A and D. We may say that instead of a Darwinian group, we are dealing with a Darwinian chain: fitness-affecting interactions occur between adjacent elements, but not between those at opposite ends of the chain. The problem is, in fact, more challenging, because this example is not abstract, but corresponds to real problems. For instance, biologists are aware of gene flow between isolated populations of sexual species. Given two populations X and Y, some individuals from population X may interact with some individuals from population Y and so impact their fitness. However, it would not mean that these two populations constitute one Darwinian population, because such relations are constrained to a number of members from populations X and Y. Indeed, members of those two populations constitute, rather, a Darwinian chain.

At first glance, such considerations might seem to be merely interesting intellectual fun, but, sadly, applicable only to certain special cases such as the one outlined above. However, if we stop focussing exclusively on the interactions between members of a single species, as in the above example, and if, moreover, we accept that Darwinian interactions might take place even between species as distant as viruses and apes, then it seems that a neighbour-structured population is the dominant, if not the only, structure in nature. We can think of many fitness-affecting interactions between species; however, generally we are inclined to think that the most important interactions take place between individuals belonging to the same species. This is intuitive and understandable; as Darwin put it a long time ago:

As species of the same genus have usually, though by no means invariably, some similarity in habits and constitution, and always in structure, the struggle will generally be more severe between species of the same genus, when they come into competition with each other, than between species of distinct genera (Darwin 1859, p. 59).

However, we can provide many examples from nature that contradict this view. For instance, Van Andel (2005) undertook a review in which he showed that fitness-affecting interactions between species (between different plant species, plant-animal, plant-fungus, etc.) are very important in shaping the structure of plant communities. This multitude of interactions provides a culture for abandoning group structure and accepting the dominance of neighbour structure in nature. Furthermore, with the inclusion of microorganisms, examples of this kind of interaction multiply. This can be seen clearly today thanks to microbial ecology, which shows that it is almost impossible to find a multicellular individual that does not engage in fitness-affecting interactions with certain microorganisms (Zilber-Rosenberg and Rosenberg 2008; McFall-Ngai et al. 2013).

What does this mean for our considerations? Let’s use an example. Every cat (I want to have a cat like Schrödinger!) interacts with a group of cats, because he competes for certain resources or for sexual partners. Furthermore, every cat interacts with a group of bacteria that occupies its gut, because bacteria produce certain secretions which, for instance, change the functionality of the cat’s digestive system. However, secretions of a bacteria from the gut of Cat A do not influence the digestive system of Cat B. Indeed, the bacteria from the gut of Cat A interacts only with certain other bacteria that live in the same gut; and what is true concerning Cat A is also true for bacteria from the gut of Cat B. We have here, therefore, a multi-species neighbour-structured population. Indeed, it seems that every bacterium and every cat has its own Darwinian population: a set of reproducers that influence its fitness. And the Darwinian population of Cat A, Cat B and selected bacteria might just more or less overlap. If we set this together with the frequency of interactions between multi-cellular individuals and microorganisms (Zilber-Rosenberg and Rosenberg 2008; McFall-Ngai et al. 2013) then we reach a simple conclusion: the world of multicellular individuals is the world of neighbour structure.

Furthermore, there is another problem tied closely to the one described above. A particular population characterised by neighbour structure need not remain in the same configuration during the lifespan of the Darwinian individuals that built it up. For instance, if we have a human being who interacts with a group of bacteria, then it is very likely that during different episodes of his life, he will interact with different microorganisms, because his food preferences and environment change, or because he gets ill. For instance, research on human microbiomes has shown that as people age, the species diversity of their symbiotic microorganisms changes (Yatsunenko et al. 2012). Therefore, it seems that neighbour-structured populations change over time, due to various perturbations.

The above examples show that even if causal interactions take place between members of a group of reproducers, it does not mean there are Darwinian interactions between all of them, or that these interactions remain the same during the lifespan of Darwinian individuals. All of this derives from the fact that populations are not permanent structures created once and for all by an artisan-like demiurge responsible for the design and maintenance of the physical universe, as Plato might have said, which might suggest that our aim as philosophers and biologists is simply to find them in nature. Rather, nature is a very dynamic structure that has been changing since the Big Bang and Darwinian populations of reproducers emerged at one point and are still dynamically changing. They change as units of selection (reproducers) multiply, evolve, and die, making the task of drawing the boundaries of a Darwinian population very hard.

Does this mean that we cannot distinguish a Darwinian group from a mere aggregation of individuals? I think we can do so; however, we have to accept that being a Darwinian population is not a property of a group but a relative property of a reproducer. I do suggest, therefore, adoption of a reproducer’s eye view—indeed, to take as a starting point not a collection of reproducers, but a single reproducer. We take a reproducer and look for other reproducers which influence its survival and reproduction and thus build the Darwinian population from the bottom up. This would enable us to avoid the problems I mentioned above, since we are building a Darwinian population through analysing the interactions of a given reproducer, thus enabling us to exclude reproducers with no impact on its fitness.

## Multi-species Darwinian populations

In the last section I treated interactions between microorganisms and multicellular individuals the way biologists usually deal with individuals belonging to the same species: if they continue to engage in fitness-affecting interaction, they constitute a Darwinian population. Unfortunately, this is not the way biologists deal with multi-species communities. I have therefore thrown out the old tradition of thinking about evolutionary phenomena, which, as Bouchard pointed out (2014), focusses on modelling interactions among entities of the same type:

Population approaches usually model the same type of entities at similar levels of organisation (cells with cells, genes with genes, individual organisms with individual organisms, groups with groups). But how are we to model evolving assemblages of motley unrelated parts at different levels of organisation?

I need to say a couple of words, therefore, to explain why a multi-species account of Darwinian population is the proper way to think about ENS and, furthermore, how to understand ENS in diverse populations built of such distinct elements as fungi, bacteria and cats.

If we start with a very basic account of natural selection—i.e. that evolution by natural selection will take place in any population (a group of causally connected objects) in which there are phenotypic variations, heritability, and differences in fitness (reproductive output) caused, at least in part, by these variations—then we have to, at least partially, abandon this tradition, because the consequence of this account is that a lion, a gazelle and a virus belong to the same Darwinian population as long as they continue to engage in causal interactions that influence their reproductive output. Godfrey-Smith (2009) calls these interactions ‘competitive interaction with respect to reproduction’, and denotes them as α (alpha), which defines the extent to which increasing one individual’s fitness reduces another’s in the population under consideration. Alpha is a continuous variable ranging from 0 to 1. The closer it is to 1, the more increasing the fitness of one individual reduces the fitness of others. In the context of the last section, this might be redefined as the extent to which increasing the fitness of a member of the Darwinian population of a given reproducer reduces the fitness of the focal unit, and so it will be understood here. For me, this parameter is a key that enables us to link a group of elements within the Darwinian population of a given reproducer despite their phylogenetic diversity. Let me present two scenarios to demonstrate this.

Suppose there is a chimpanzee that competes with a group of other chimpanzees for a limited number of bananas. Suppose, then, that the banana is a crucial resource that is necessary to survive and reproduce. Suppose as well that other resources, such as sexual mates, etc., are unlimited. Our chimpanzees, therefore, have no problem acquiring resources other than bananas. In such a scenario, therefore, competition would involve just one resource and differences in fitness would mirror differences in acquiring bananas. Suppose that, in a second scenario, a chimpanzee competes with a group of chimpanzees and cats; for both—cats and chimpanzees—bananas are a crucial resource and all other resources are unlimited. Again, therefore, competition would involve just one resource and differences in fitness would mirror differences in acquiring bananas. Cats and chimpanzees that are able to acquire them readily would increase in number over generations, and in the end a particular phenotype of a cat or a chimpanzee (one that knows perfectly well how to get bananas) might outcompete other chimpanzees and cats. These scenarios are, of course, fictional. However, they show an important thing: in both scenarios there is strong competition over resources that leads to the diverse reproduction of individuals. It does not matter whether they belong to the same species or not, because their reproductive output is limited. Their fitness is interdependent: the more offspring I produce, the less you are able to produce; or, as Godfrey-Smith (2009) wrote, ‘a slot I fill in the next generation is a slot that you do not fill’. This might be easily understood when there is a strong competition among conspecifics. However, the same goes for the competition between viruses and cats. Of course, they do not compete for bananas directly like the cats and chimpanzees in the scenario above. For instance, if we were to add viruses to that scenario, then they would compete for bananas as well, but in a more abstract sense. Viruses would try to use certain host resources (such as necessary nutrients that the host has assimilated from bananas) for production of their own offspring. Indeed, viruses would use resources that the host might have used to multiply. Thus, a slot that might be filled by host offspring would be filled instead by viruses.

So far we have been considering only one side of the coin: namely, fitness-affecting interactions with a negative impact on the fitness of the focal unit. However, when we take a reproducer and look for the other reproducers making up its Darwinian population, then we see that it engages as well in interactions exerting a positive impact on fitness. I call these interactions *fitness*-*enhancing*. An interesting question is whether their existence is necessary for evolution by natural selection to occur in multispecies Darwinian populations. I think the answer is ‘no’. I think that competitive interactions are more primal and fundamental than enhancing interactions, and that the latter, in fact, evolve in response to the former. As Nowak (2006) put it: ‘The question how natural selection can lead to co-operative behaviour has fascinated evolutionary biologists for several decades’. That said, co-operative interactions are considered much more complex, and a fundamental issue in evolutionary biology is to understand the conditions under which they can evolve from competitive interactions. To give an example of such conditions: in algae, single cells co-operate to form clusters in response to competitive interactions with predatory protists, because this strategy reduces their chance of being eaten (Boraas et al. 1998; Fisher et al. 2016).

In spite of this, I think that for natural selection to occur, competitive interactions with respect to reproduction are sufficient; it is from them that fitness-enhancing interactions derive. In other words, when there is strong competition between Darwinian individuals, some of them might engage in co-operative actions with others in order to enhance their common fitness. This kind of interaction might have a positive influence on the fitness of the focal unit and change the outcome of competitive interactions. Thus, in many cases, the Darwinian population of a given reproducer would comprise individuals exerting both a positive and negative influence on its fitness; the reproductive output of the focal unit would be the result of these two types of interactions. However, they are not necessary, either for the process of natural selection to occur in multispecies populations or for the subsequent parts of this paper; thus, while interactions of this kind are very interesting, I am not going to consider them more deeply.

However, Matthewson (2015), and Godfrey-Smith as well (2009), argued that this is not the whole story (i.e. alpha) and that something more is needed in order to have a population that might undergo natural selection. Building upon Templeton’s (1989) idea of species, Matthewson concluded that a group of individuals engaged in fitness-affecting interactions must be under the influence of mechanisms that sustain their similarity in order to undergo paradigm natural selection. Thus, he argued that we need another parameter, which he called exchangeability and divided into genetic and demographic. The former refers to the ability to combine genes with others in the group, the latter to a situation in which individuals occupy the same niche and thus are under the same selection pressure. These additional mechanisms, if strong, assure that the individuals in question are very similar. Matthewson’s intention in introducing this new parameter was to avoid situations in which a group of individuals is called a Darwinian population simply because they have a high alpha value, when, in fact, they compete over just one crucial resource and have nothing else in common. That’s right: they might be just two different species, occupying niches that, despite one similarity, are completely different. For instance, a chimpanzee and a virus. As a result, he argued, natural selection would not lead to a situation in which fitter individuals take over a niche, as it is hard to imagine a virus taking over a chimpanzee’s niche.

Well, I agree this is true, but, at the same time, I do not think that this is what natural selection is about. Generally, evolution by natural selection concerns causative interactions that lead to differences in the reproductive output of individuals struggling for existence, as I argued above. Of course, very often evolution by natural selection leads to a niche being taken over by fitter individuals. This is the case when there are strong completive interactions among members that exchange genes, as in sexual species in which a fitter allele may become fixed in a gene pool. Sexual reproduction may even be very important, because it ‘speeds up’ the formation of new combinations of alleles, and cumulative adaptation might emerge much faster within such a group of individuals (Morran et al. 2011). However, I don’t think that exchangeability states *whether* there will be natural selection; rather, it is just another parameter (albeit an important one) indicating what the evolution of a population under consideration would look like. For instance, given a high rate of exchangeability, we might see how one phenotype takes the place of another in a given niche, as when a bacterial strain evolves a new trait that enables it to acquire resources more rapidly and, as a result, outcompete other strains. However, given a low rate of exchangeability, Darwinian selection might still exist on the highest level. For example, during the 1918 flu pandemic the rate of exchangeability between the flu virus and human beings was low; nevertheless, the reproductive output of many people was shaped mainly by interactions with viruses.

Basically, I agree that exchangeability is an important parameter. However, I do not think that it should be placed on the same level in the hierarchy as α, because alpha is the parameter that decides whether or not there is a struggle for existence. If alpha is high, then the individuals in question engage in Darwinian interactions despite their low rate of exchangeability. However, if there is a high rate of exchangeability but very low alpha, then there is no Darwinian selection, but only a group of individuals that are similar because they occupy the same niche and/or can potentially interbreed. Thus, I think that the Darwinian population of an individual is determined by interactions that influence its reproductive output. The stronger these interactions, the more there is a struggle for existence, and the greater the likelihood that a population is Darwinian. Other factors such as exchangeability, fitness enhancing interactions, variance, integration, etc. are secondary characteristics of a Darwinian population that might help us understand how the community in question will evolve.

## Fitness incommensurability

I think you, the reader, might be puzzled right now, because if you accept the statement that natural selection is taking place between phylogenetically distinct species, than an obvious problem emerges: how to compare the fitness of a virus and a cat when they are engaged in strong interactions that influence their reproductive output? This is, I believe, a major problem that turns people away from the multi-species Darwinian account of population. This is because natural selection is tightly linked with the concept of fitness and this linkage dictates the way scientists think about evolutionary phenomena. I believe it goes like this: if there is natural selection, then there are differences in reproductive output, and if there are reproductive differences then we can quantify and compare them and say who is fitter. However, if we cannot compare them, then there is no natural selection, since in that case, how can we say who is fitter?

Such logic creates the illusion that fitness and natural selection are very related concepts. Indeed, it suggests that we cannot have natural selection unless we can quantify fitness differences. I think that the first step, therefore, toward acceptance of the multispecies account of a Darwinian population is to separate fitness from natural selection, and to demonstrate that we can have the process of natural selection without being able to make any claims about fitness differences. Thus, let me add a couple of words about fitness, in order to provide a background to help explain how to understand fitness in multispecies Darwinian populations. Philosophical (such as ‘what is?’) and practical questions (such as ‘how to count?’) concerning fitness are very complex and have been debated for decades (Abrams 2012; Bourrat 2015). However, I put them aside here, because for the purpose of this paper a few general remarks are sufficient.

Thus, let me start with a fundamental question: who or what is the bearer of fitness? This is not a trivial question, given that fitness is not very well defined, and has been ascribed to different types (or even groups, such as a given phenotype) of entities (Abrams 2012; Bourrat 2015). My answer to this question will not be surprising, since I have already answered it indirectly in this paper. The bearer of fitness is simply a Darwinian individual, a unit adapted to the reproduction of itself. It might be a simple bacterial cell or a complex collective reproducer such as a cat. Even though collective reproducers are built of elements that are themselves capable of reproduction (such as multicellular individuals built of eukaryotic cells), I am inclined to argue that the bearer of fitness is a collective reproducer, because the cells that make up a cat are adapted to the cat’s reproduction, and thus function in a coordinated way to make more cats, as Michod (2009) put it: “By specializing, cells relinquish their autonomy in favor of the group; as a result, fitness and individuality are transferred from the level of the cell to the level of the group.” The exception would be cancer cells, which are adapted to reproduce themselves rather than the collective reproducer they are part of; however, they are irrelevant to the purposes of this paper. Thus, in this context, fitness differences simply mean differences in the reproductive output of Darwinian individuals.

This is the way we usually think of fitness differences when discussing fitness. For instance, if we take two cats that are in reproductive competition, and one has more offspring than the other, then we tend to say that the first cat is better fitted to the environment (has a higher level of fitness), because it copes better with environmental factors, which results in its having more offspring. On the other hand, when we see a virus that infects a cat and has a lot of offspring, while the cat, which, due to the presence of this virus, has none, we are not really inclined to say that the virus is fitter than the cat. Why not? After all, in both scenarios we have reproducers engaging in competitive interactions which affect their reproductive output.

The reason is that fitness is supposed to give us information on how well particular individuals are adapted to a particular environment (Dennet 1995;Bourrat 2015). Unfortunately, a virus and a cat, although they may engage in fitness-affecting interactions, are adapted to completely different environments. Thus, it makes no sense to compare their reproductive differences, because their fitness is incommensurable. This basically means that we should not judge their reproductive outputs by the same standards, since they are an effect of totally different environmental factors (ranging from generation time through sexual selection to other abiotic/biotic factors) to which both cat and virus have been adapting for many generations. Indeed, for the former, producing two offspring is a success, while for the latter, a thousand offspring would not qualify as a success. Hence, comparing their reproductive success is pointless and inconclusive, since they have adapted to totally different environments. In other words, in order for us to compare the fitness of two units, they must be subject to very similar selective forces. I think, therefore, that the idea of exchangeability may be a good measure of fitness commensurability, because if there is a high rate of exchangeability, the individuals in question are subject to the influence of mechanisms that sustain their similarity and so plausibly occupy the same niche (abiotic and biotic), i.e. they are under the same selective pressure.

The idea of fitness incommensurability provides us with a basis for separating fitness from the process of natural selection. On one hand, natural selection is a process that leads to different reproductive outputs of reproducers engaged in fitness-affecting interactions. On the other, fitness is supposed to determine which individual is better adapted to a given environment. These two ideas are very useful as long as we are dealing with subjects with a high level of exchangeability, such as two cats that engage in fitness-affecting interactions: here we have two units that are undergoing evolution driven by natural selection, and we can say which one is fitter. However, these two ideas are not as useful when we have two units characterised by a low level of exchangeability, such as a cat and a virus. They might both be subject to strong selection, since the virus might be extremely virulent and thus might influence the fitness of the cat, but at the same time have a very low rate of exchangeability. Therefore, such interactions, even though they are extremely important, do not permit us to legitimately compare the fitness of a host and a pathogen, since they are adapted to different environments (both biotic and abiotic) and thus their fitness output is not comparable. The conclusion, therefore, is that we can have evolution driven by natural selection, and yet be unable to draw any conclusions about fitness differences, since units that engage in Darwinian interactions differ substantially.

I think that my use of Matthewson’s (2015) idea of exchangeability constitutes a useful refinement of the ideas of Godfrey-Smith (2011), which he presented in his discussion on multispecies interactions. Godfrey-Smith (2011), in his response to Sterelny’s criticism (2011), argued that, in the case of multispecies combinations such as acacias and ants, we should distinguish two Darwinian subpopulations, each of which operates as part of the other’s environment. As the reader might deduce from the previous section, I do not agree with this distinction, since, as I have argued, there are no good theoretical reasons not to have multispecies Darwinian populations. Indeed, Darwinian interactions may take place even between phylogenetically distinct species. Thus, what Godfrey-Smith distinguished were not Darwinian groups but groups of commensurable individuals, or groups characterised by high rates of exchangeability, which enables us to draw conclusions about fitness differences within them. However, I still find this division useful for practical reasons. This is because a good part of science comprises research which attempts to determine which individuals are better adapted to a given environment. Thus, singling out individuals with commensurable fitness and treating the rest as an environment is a useful simplification that might serve many research purposes.

My conclusions, therefore, concerning fitness in multi-species populations are different than those put forward by Bouchard (2014). He suggested that in the case of multi-species communities we should abandon reproductive output as a measure of fitness and focus on the persistence of communities as a better measure. I think this is unnecessary, provided we accept our inability to compare the fitness of all elements of such communities. While I do not discount the possibility that persistence may be a good long-term indicator of fitness, I think that, within populations, reproductive output seems to be the most practical approach. In the next section I will show how it (together with other concepts from this paper) might be useful to understand the ecology of endosymbiosis

## Ecology during the origin of endosymbiosis

One of the most interesting issues in evolutionary biology is our understanding of how collective reproducers such as multicellular individuals or eukaryotic cells evolved from lower ones (Buss 1988; Maynard-Smith and Szathmary 1995; Okasha 2006; Godfrey-Smith 2009). Why did lower-level units come together to form a higher unit? Why didn’t selection at the lower level disrupt the functionality of the higher unit? To solve these and many other relevant issues, researchers have made extensive use of concepts that were primarily developed to study ecological issues, such as cooperation, mutualism, conflict resolution, etc. This was a fairly useful approach, leading to the expansion of our knowledge and I believe it will continue to be useful in future.

At the same time, however, it seems that it is sometimes hard to extrapolate concepts from ecology, because it is not clear how to do so. One paradigmatic case is the origin of cellular organelles like mitochondria or chloroplasts. While it is very easy to say, as some do (Margulis 1981; Sachs and Simms 2006), that symbiosis between a host cell and an endosymbiont is an example of cooperation, because both participants benefit (i.e. the host obtains certain products and the bacteria reveives protection), understanding the origin of this symbiosis in ecological terms is much more complex. This derives from the absence of free-living organelles; thus, as Maynard-Smith and Szathmary argued in their seminal work The Major Transitions in Evolution (1995), we cannot measure their fitness and compare it with that of mitochondria. However, I will go even further here and say that even if someday we were to find a free-living mitochondrion (whatever that might be), measuring its fitness would not enable us to decide whether the transition from a free-living state to life as an organelle was beneficial to the bacterium or not, because this question makes no sense. Why not? Let’s try to look at this ancient event from the perspective of the bacterium that was engulfed.

Suppose there is a bacterium that competes with two other bacteria for limited resources. Suppose as well that there is one parasite. If the parasite makes our bacteria a host, then it is reducing the fitness of the bacteria, because it may curb its abilities and reduce its acquisition of resources compared to the other two bacteria. As a result, the other two bacteria can multiply at a higher rate. However, if the parasite engulfs our bacteria and transforms it into an organelle, then it automatically changes our bacteria’s Darwinian population. Indeed, the bacteria no longer competes for resources with the other two bacteria, but may, for instance, compete with other parasites for hosts. Indeed, it is placed under different selection pressure. Asking, therefore, whether endosymbiosis reduced or decreased the fitness of endosymbionts compared to free-living bacteria is pointless, because following this event the fitness of free-living bacteria and endosymbionts is incommensurable. That` because an indicator of a high level of fitness prior to endosymbiosis (such as a high rate of multiplication or defense against predators, etc.) might be meaningless after endosymbiosis and, alternatively, an indicator of a high level of fitness following endosymbiosis (such as decreased virulence) might have been of no significance previously. Comparing levels of fitness (by measuring rate of growth, etc.) would thus be pointless and inconclusive in light of the completely different environments to which the individuals in question have adapted. Indeed, from the perspective of a free-living bacterium, it may be a good thing to multiply, but from the perspective of an endosymbiont it may lead to damaging the host and thus to reducing its fitness; since they are now reproducing as a whole, their evolutionary ‘success’ is linked (Leigh 1991; Stencel and Crespi 2013).

At first glance the above considerations seem to be correct. Unfortunately, there is still a flaw in the logic. Contrary to Maynard-Smith and Szathmary (1995), I do not think that there is, in fact, any sense in comparing the fitness of an organelle with that of a free-living bacterium from a sister lineage. This is because following endosymbiosis, as I pointed out, an organelle becomes part of the collective reproducer. That’s right: an organelle and its host cell constitute one Darwinian individual that is undergoing evolution as a whole by means of natural selection. Therefore, we have to compare the exchangeability of this entire new unit with the sister lineage of the endosymbiont to see whether we can say something about the superiority of this newly evolved unit over the free-living ancestors of the endosymbiont in terms of fitness. And since the origin of life there have been many different scenarios; thus, looking at this issue from the perspective of the collective reproducer is much more complex than from the organelle’s perspective. Let’s have some fun picturing this diversity of endosymbiosis and consider two plausible scenarios.

One of the most ancient episodes of endosymbiosis is the evolution of a protocell from free-living replicators encapsulated inside a membrane, whereby a new unit composed of lower-level elements emerged (Maynard-Smith and Szathmary 1995). The advantage of this transition was that the replicators inside the cell were linked and could perform a variety of tasks. Indeed, each free-living replicator had to perform all tasks in order to reproduce, while a group inside a ‘bag’ could divide labour and perform tasks more effectively and, as a result, outcompeted free-living replicators. In this scenario we could have compared—with a large degree of probability, I think—the fitness of free-living replicators with the fitness of those encapsulated, because they were adapted to the same environment and some of them just ‘decided’ to jump into a ‘bag’ without leaving their niche. Thus, it is very likely that the degree of exchangeability of the ‘replicator bag’ and a free-living replicator was very high back then.

The above example of endosymbiosis is characterised by the fact that the units that were undergoing transitions in individuality were characterised by a high degree of exchangeability at the very beginning. However, endosymbiosis sometimes involves agents with a low degree of exchangeability. My favourite example is endosymbiosis between the bacteria *Buchnera sp.* and aphids (Baumann 2005). In this scenario it is very likely that the fitness of the newly-evolved unit would be incommensurable with that of a free-living bacterium, because it is part of a collective reproducer that competes currently with different reproducers. Indeed, the newly-evolved unit (the aphid that the bacterium is part of) does not compete with the same units as a free-living bacterium, but is currently under different selective pressure. However, this is not the whole story concerning this ancient event. So far, we have been considering only the endosymbiont’s perspective. If we zoom in on the Darwinian population of the host that engulfs our bacteria, then we will draw different conclusions. Following endosymbiosis, the host, as opposed to the endosymbiont, abruptly changes not its Darwinian population, but its inner structure. Indeed, the host cell is still competing with the same reproducers, but at the same time it acquires a lot of new genes, raw material for natural selection to work on.

Of course, I have presented only two scenarios of endosymbiosis and there may be many other possibilities. For instance, the exchangeability of the collective reproducer might differ from that of both the host and endosymbiont, because their fusion might lead to the emergence of a new trait which enables it to occupy a new niche. However, my aim is not to give a review of plausible scenarios, but to show that we may think of the origin of endosymbiosis in more precise terms than using poetic metaphors such as ‘slavery’ (Maynard-Smith and Szathmary 1995), which are very beautiful, but have no epistemological content. Alternatively, by thinking of a Darwinian population as a property of a Darwinian individual, we might follow the paths of agents that are undergoing endosymbiosis and understand how their Darwinian populations change during that event, i.e. whether the exchangeability of the agents in question changes compared to that of agents that did not undergo endosymbiosis. Understanding this, let us ask questions about the adaptivity of endosymbiosis. For instance, it might happen that the exchangeability of agents does not change much during this process (recall the origin of the protocell) and the fitness of a collective reproducer is still commensurable with that of free-living entities from a sister lineage; thus, we can draw conclusions about the fitness superiority or inferiority of the newly-emerged reproducer. Alternatively, in other situations, the exchangeability of the unit that came into existence might change so much that their fitness would not be commensurable, at least for one of the participants of endosymbiosis (recall the symbiosis of the aphid and the bacterium).

The conclusion is that after endosymbiosis, the exchangeability of one agent (or both) might change so much that it would make no sense to compare its fitness with members of the sister lineage. However, as I pointed out in the last section, they might still engage in fitness-affecting interactions. This would happen if a parasite were to become part of the host and find itself under pressure to evolve an effective immune system, while members from the sister lineage were under pressure to overcome the immune system of the host. Their exchangeability has changed drastically, but they are still engaged in fitness-affecting interactions; one is part of the environment of the other and vice versa. We cannot compare their fitness, but it may be still interdependent, provided they continue to engage in fitness-affecting interactions; thus it might happen that ‘(…) a slot I fill in the next generation is a slot that you do not fill’ (Godfrey-Smith 2009).

## Concluding remarks

Recent discoveries in the field of microbiology have been systematically transforming the way we see the biological world surrounding us (O`Malley 2014). One of the most astonishing discoveries was the finding that living individuals that we had perceived as belonging to well-defined categories, such as plants or animals, can function properly only in the presence of symbiotic microorganisms, which may be necessary for the development of immune systems (Mazmanian et al. 2005) or digestation of necessary nutrients (Ley et al. 2006) and may even influence mating preference (Sharon et al. 2010). Thus, viewing living objects as mosaics of hosts and microorganisms is no longer controversial. In this context, Dupré and O’Malley (2009), using a framework developed by Hull (1980), argued that we should consider such a collaboration of host and microorganisms engaged in a network of biochemical interactions to be ‘the most fundamental unit of selection’. In this paper, I have taken another approach: using Godfrey-Smith’s (2009) elaboration of natural selection, I have argued that each unit that reproduces as a whole should be considered a unit of selection, and the remaining units that influence its fitness should be counted as members of its Darwinian population, which may be a multispecies unit. Thus, in this context, a microbe and a host which are engaged in a network of biochemical interactions but which do not reproduce as whole do not constitute a unit of selection, but rather two independent units engaged in a biochemical network. This is because, for instance, microbes were for many generations part of the host’s Darwinian population, and the host may have adapted through making good use of the microbes’ biochemical machinery. Thus, my paper offers an alternative way of looking at the host-microbe relationship.

This paper contributes to the solution of another major problem: understanding the role of natural selection in the evolution of collective reproducers, such as multicellular individuals or eukaryotic cells that have evolved from lower cells (Buss 1988; Maynard-Smith and Szathmary 1995; Okasha 2006; Godfrey-Smith 2009). A common question is: why did lower-level units come together to form a higher unit? A common approach to answering this question is to look for the adaptive advantages of such a transition—in other words, to find selective forces which would allow natural selection to choose collective reproducers over simple ones and so promote a transition to a collective ‘lifestyle’. To recall, in algae, single cells co-operate to form groups in response to competitive interactions with predatory protists, because it reduces their chance of being eaten (Boraas et al. 1998; Fisher et al. 2016). Recently some researchers have suggested that the early stages of such a transition (formation of a group) might occur without the participation of natural selection (Fleming 2012; Fleming and Brandon 2015). As Fleming and Brandon (2015) put it, ‘Groups are expected to form based on the underlying tendency of evolutionary systems to increase in variance’. My work on endosymbiosis shows that the formation of a group may sometimes be non-adaptive, at least for one of the participants. For instance, when one single cell engulfs another, this may be adaptive for the host cell, as it acquires a lot of new genes—raw material for natural selection to work on—which may provide immediate benefits for the host. This, as Haynes argued (1990), can therefore be considered, from the perspective of the host cell, a mega-mutation. However, from the perspective of the engulfed unit, it may be strictly a non-adaptive process. This might occur when endosymbiosis changes the unit’s Darwinian population entirely and when, following endosymbiosis, it has to compete with different individuals. Indeed, in such a situation an endosymbiont is taken out of competition with the members of its old Darwinian population and acquires new competitors. Therefore, to draw a parallel with card games, we can say that endosymbiosis, for such a unit, resembles shuffling a deck of cards (a Darwinian shuffle): an endosymbiont is dealt a new hand and the game starts again. Natural selection then rejoins the game and selects the most optimal combinations of host and endosymbiont. Thus, my work shows that during evolutionary transitions there may sometimes be room for the co-operative play of chance and natural selection, but this need not always be the case (recall the evolution of the protocell).

Assuming that Darwinian populations are relative properties of reproducers is a very fruitful approach which enables me to understand how to set the boundaries of multispecies Darwinian populations and to provide a novel understanding of the ecology of endosymbiosis. However, even though I am convinced that this is the proper way to think about Darwinian populations, I am aware that this approach might not be particularly useful in connection with certain other scientific issues. For instance, if one wishes to study the dynamics of multispecies communities, constituting hundreds of individuals belonging to many different species, in order to understand how they influence the pedosphere, then focussing exclusively on the Darwinian population of a given reproducer might obfuscate the bigger picture. As Bruce Lee put it in *Enter the Dragon*: ‘Don’t concentrate on the finger or you will miss all that heavenly glory’. This is because some important changes in the pedosphere may be caused by the interactions of microbes that do not engage in fitness-affecting interactions (such as geochemical cycles), but instead, for instance, remain at the extremes of neighbour structure (Fig. 1b). One good way not to miss ‘all that heavenly glory’ is to try to visualise these networks of interactions so as to include interactions of different kinds. Such a project is very ambitious and beyond the scope of this paper. The first steps in this direction, however, were taken by Bapteste and Dupre (2013), who attempted to provide such a multidimensional framework taking into account many different types of interactions. I think this is a valid direction and that biology will gradually move toward it in order to attain a deeper understanding of the factors shaping the diversity of the biosphere.
